# A critical analysis of the implementation of service user involvement in primary care research and health service development using normalization process theory

**DOI:** 10.1111/hex.12237

**Published:** 2014-07-24

**Authors:** Edel Tierney, Rachel McEvoy, Mary O'Reilly‐de Brún, Tomas de Brún, Ekaterina Okonkwo, Michelle Rooney, Chris Dowrick, Anne Rogers, Anne MacFarlane

**Affiliations:** ^1^ Graduate Entry Medical SchoolUniversity of LimerickLimerickIreland; ^2^ Discipline of General PracticeNational University of IrelandGalwayIreland; ^3^ Galway Migrant ServiceGalwayIreland; ^4^ Mayo Intercultural ActionBallinaIreland; ^5^ University of LiverpoolPrimary Medical CareLiverpoolUK; ^6^ NIHR Collaboration for Leadership in Applied Health Research and CareWessexUK; ^7^ Faculty of Health SciencesUniversity of SouthamptonSouthamptonUK; ^8^ Primary Health Care ResearchGraduate Entry Medical SchoolUniversity of LimerickLimerickIreland

**Keywords:** critical interpretive synthesis, normalization process theory, patient participation, primary health care, service user involvement

## Abstract

**Background:**

There have been recent important advances in conceptualizing and operationalizing involvement in health research and health‐care service development. However, problems persist in the field that impact on the scope for meaningful involvement to become a routine – normalized – way of working in primary care. In this review, we focus on current practice to critically interrogate factors known to be relevant for normalization – definition, enrolment, enactment and appraisal.

**Method:**

Ours was a multidisciplinary, interagency team, with community representation. We searched EBSCO host for papers from 2007 to 2011 and engaged in an iterative, reflexive approach to sampling, appraising and analysing the literature following the principles of a critical interpretive synthesis approach and using Normalization Process Theory.

**Findings:**

Twenty‐six papers were chosen from 289 papers, as a purposeful sample of work that is reported as service user involvement in the field. Few papers provided a clear working definition of service user involvement. The dominant identified rationale for enrolling service users in primary care projects was linked with policy imperatives for co‐governance and emancipatory ideals. The majority of methodologies employed were standard health services research methods that do not qualify as research with service users. This indicates a lack of congruence between the stated aims and methods. Most studies only reported positive outcomes, raising questions about the balance or completeness of the published appraisals.

**Conclusion:**

To improve normalization of meaningful involvement in primary care, it is necessary to encourage explicit reporting of definitions, methodological innovation to enhance co‐governance and dissemination of research processes and findings.

## Background

The idea of involving patients and the public in health care has grown significantly in recent decades and is now enshrined in health policy across a range of international settings.^1^, ^2^, ^3^, ^4^, ^5^

Therefore, patient and public involvement (PPI) has increasingly become the focus of attention in health services research and health services development. Thompson *et al*. argue that these are overlapping categories whereby data generated by such research can inform and improve health‐care services. There are a number of models or frameworks that aim to conceptualize public and patient involvement (PPI). Gibson *et al*.'s^7^ recent work on conceptualization of PPI provides a valuable overview of models and frameworks in the field, and provides new theoretical directions (that were previously absent) for an emancipatory concept of patient and public involvement in health services development. A systematic review by Brett *et al*.^9^ focused on the conceptualization, measurement and impact of outcomes of PPI in health and social care research.

However, conceptualization and theorization of PPI is not common in studies of PPI.^9^ Furthermore, there have been recent important developments in the operationalization of public and patient involvement in health research and health service development. Earlier literature in the field proposed that involvement could be captured through the use of conventional health service research methods such as surveys, in‐depth interviews and general consultation.^10^ It is now accepted that these methods, by themselves, do not facilitate meaningful involvement unless service users have contributed to research design. This emphasis on more extensive involvement is captured in the www.involve.org.uk INVOLVE definition of involvement in health research and health service development as research ‘with’ or ‘by’ service users, rather than ‘to’, ‘about’ or ‘for’ service users (see www.invo.org.uk).

There have been examples of concerted efforts to develop infrastructure and capacity to support meaningful collaborations and partnerships between academia, health‐care providers and patients in North America (see http://pram.mcgill.ca/) and the United Kingdom (see http://piiaf.org.uk/).

However, problems persist in the field. Firstly, there is a problem of definition. There is still a wide range of terms used in the field, including patient involvement, patient engagement, patient participation, service user involvement, citizen engagement, community participation, community engagement and public involvement.^11^ Gallivan *et al*.^12^ argue that this can contribute to misunderstanding and misinterpretation of expectations, goals and outcomes by different groups of stakeholders, which poses barriers to achieving meaningful and successful outcomes in partnership work together. We clarify our terminology in Box 1.

Box 1 **Terminology: service user involvement**

In this paper, we employ the term Service User Involvement (SUI) because this is the terminology employed by the Health Service Executive (HSE) in Ireland in its Strategy for Service User Involvement 2008,^6^ and the research reported here is designed to inform our national policy context as well as informing international debates. In the Irish context, the term SUI refers to ‘a process by which people are enabled to become actively and genuinely involved in defining the issues of concern to them, in making decisions about factors that affect their lives, in formulating and implementing policies, in planning, developing and delivering services and in taking action to achieve change’.^3^, ^6^ The term SUI was chosen from a variety of options (e.g., engagement, public participation and community participation)^4^, ^5^, ^8^ as a workable rather than ‘perfect’ definition.

Secondly, there are many reasons why service users and health professionals get involved with service user involvement (SUI) projects. This presents the problem of enrolment in the field.^6^, ^13^, ^14^, ^15^ We do not know what factors motivate health professionals or service users to enrol in specific projects: Is it a question of personal motivation or is it a response (voluntary or involuntary) to policy directives? Are service users and health professionals enrolling with shared or differential motivations and definitions of involvement?

Thirdly, there is the problem of enactment. As above, standard conventional research methods can be mistakenly conflated with SUI.^16^ It is important to know why a specific method is selected for a project and whether the selected methods are *congruent* or not with an intended level of involvement and working definitions of service user involvement in health research and/or health service development.

National and international literature reviews of the field have highlighted that it is very possible for health‐care professionals to be ‘engaged’ in numerous purported involvement activities with service users *without genuinely involving people* (particularly if the professionals continue to set and drive the agenda and make decisions about services and treatments without involving service users in a meaningful way).^8^, ^17^ This has implications for understanding the outcomes of SUI, which is the fourth problem – appraisal of SUI. While negative effects of SUI on research processes and subsequent health service outcomes have been reported,^9^, ^18^, ^19^ there is growing evidence that participatory approaches to research that involve service users in a meaningful and sustained way can have positive impacts in terms of setting the research agenda, programme sustainability and advancement, the generation of systemic change,^17^, ^20^, ^21^, ^22^ and on service users themselves.^23^, ^24^, ^25^, ^26^ Therefore, we have to seriously and critically analyse any claims about outcomes based on SUI where, in fact, service user involvement did not occur or was so poorly enacted that it ought not to be claimed as genuine service user involvement.

Overall, our observation is that the four problems outlined above are problematic in and of themselves, but they are also barriers to the implementation of meaningful SUI as a routine way of working in health‐care research and health‐care service settings, that is as a normalized practice. The problems are about the definition, enrolment, enactment and appraisal of SUI, and these resonate with the four constructs of Normalization Process Theory (NPT)^27^ (see Table 1). This is a contemporary social theory that can be used to understand and investigate the normalization of innovation in health care.^28^

**Table 1:** SUI and normalization process theory^27^

Question pertaining to SUI	Problems in the practice of SUI	NPT construct
How is service user involvement defined?	Definition	Coherence
Why do stakeholders get involved?	Enrolment	Cognitive Participation
What methods are used?	Enactment	Collective Action
What are the outcomes?	Appraisal	Reflexive Monitoring

The aim of this review was to critically interrogate the conditions for the implementation of SUI in both primary care research and health service development projects to make recommendations that will enhance chances of its normalization. We focus on a sample of original published empirical work that is reported as SUI in the primary care literature to rigorously examine the way definition, enrolment, enactment and appraisal are reported vis‐à‐vis each other.

## Methods

The methodological approach for conducting this review followed the broad precepts of a critical interpretive synthesis (CIS).^29^ We employed an inductive and iterative approach using the research question as a compass during the review process. We sought a purposeful sample of papers, integrated quantitative and qualitative data, and aimed for a more fundamental critique of literature (rather than a summary). While we adopted an inductive approach at the outset of the review process, given our interest in implementation and normalization, we used NPT as a heuristic device to synthesize emergent findings and draw out key recommendations.

The research team constituted academics, health authority personnel, clinicians and community organization representatives, all of whom have experience of using participatory research approaches.

**Figure 1:**
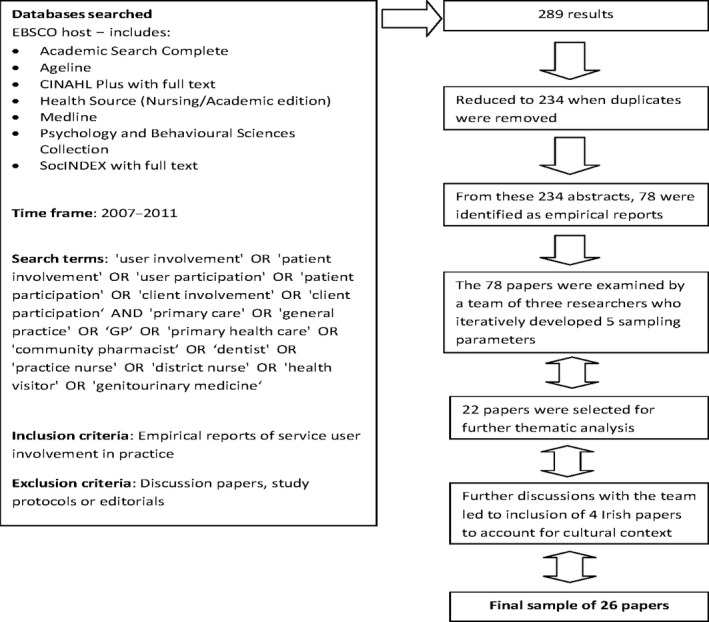
Sampling and selection process for papers included in the critical review.

We searched EBSCO host for original primary care papers about research and health service development projects that were identified by relevant search terms between 2007 and 2011 (see Fig. 1 for description of search terms).

The first stages of the review involved an iterative reflexive approach to searching and sampling the literature using a series of identification, sampling and appraisal steps as per the CIS methodology. Each of these stages of the review was led by the first author with substantial input, independent reviewing of abstracts and development of sampling parameters by the second and last author, and consultation with the other authors. The thematic analysis and subsequent synthesis of findings was led by the first and last author in consultation with all other authors, using a combination of data analysis clinics, project meetings and email correspondence.

We identified 289 abstracts at the outset, which resulted in 234 abstracts after duplicates were removed. From this, 78 empirical abstracts were identified, and using iteratively developed sampling parameters, we excluded conventional qualitative research studies and selected a final purposive sample of 26 papers for inclusion in the review (see Fig. 1).

Not all papers included in the review involved service users in a meaningful way as per the definition espoused by INVOLVE, but we included them on the basis that they were identified in the literature as SUI. Our search terms therefore represented ‘current practice’ reported in peer‐reviewed published literature. They were included because they contained at least some data about definition or enrolment or enactment or appraisal of SUI. For example, the work was presented as SUI in the introduction section against the policy background of SUI, or the work reported revealed proximity between data generation and health‐care service outcomes, which we considered relevant to understanding the impact and appraisal of SUI.

Table 2 provides an overview of the final set of sampling parameters and the numbers of papers reviewed per parameter. Following Dixon Woods,^29^ and using checklists developed by others,^30^, ^31^ we conducted a quality appraisal exercise on these 26 papers with an emphasis on the *relevance* of the paper to our review. All were deemed appropriate for inclusion in the review.

**Table 2:** Sample of 26 papers included in the critical review categorized by six sampling parameters

*n *= 8 – SUI studies explicitly reporting experience of ‘doing service user involvement’ and/or studies that demonstrate high‐level involvement using participatory methodologies
*n *= 3 – Qualitative and quantitative health services research (HSR) studies that focus on the perspective or experiences of service users, with more of an emphasis than other HSR studies on reporting outcomes or actions taken as a result of their input
*n *= 5 – Qualitative and quantitative health services research studies on the theme of SUI and/or patient participation
*n *= 2 – Studies with a focus on shared decision making, including studies that analyse patient/practitioner consultations in which there is shared decision making
*n *= 4 – Randomized controlled trials where the intervention component has some evidence of patient involvement, for example learning skills for self‐management, participation in mentoring or coaching
*n *= 4 – LLIrish papers (including grey literature) focusing on user involvement in primary care in the Irish context, as recommended by our research team to ensure our review (a) had relevance to the national policy context and (b) was inclusive of studies with participatory methodologies, which were under‐represented at one point in the sampling process

For the data extraction process, we used a modified version of the critical appraisal tool (CAT).^32^ Our process and working definitions for the synthesis are shown in Box 2. First‐order constructs were drawn from the information extracted during the critical appraisal process and were informed by the items on the amended CAT^32^ (see Table 3, Column 1). All papers, qualitative and quantitative, were appraised by the same method.

**Table 3:** Translation to inform first‐order, second‐order and third‐order constructs and their arrangement in temporal sequence

First‐order constructs informed by CAT Wright *et al*.	Studies	Second‐order constructs – emergent inductive themes	Studies	Third‐order constructs following NPT 27 May and Finch 2009	Studies (References in bold indicate quantitative papers)
1. Socio‐political context or drivers	52 33, 49, 66 **(** 34, 50 **) (** 36, 39 **) (** 67 **)** 42 **(** 35 **)** 41 68 47 46 45, 53 69 38, 56	1. Control and Power	33, 34, 35, 41, 45, 46 **(** 38, 43, 50, 52, 69 **)** 56, 68 37, 40, 44, 55	1. Definition	40, 41, 42, 43, 44, 45, 50, 55 33, 34, 70 **(** 36 **)** 37 38 51 43
2. Definition of service user involvement	**(** 35 **) (** 34, 36, 38 **)**	2. Dialogue and communication	33, 41, 45 39, 43, 69 **(** 36, 49, 50, 51, 52, 53, 56, 66, 67, 68 **)**	2. Enrolment	**(** 39 **)** 40, 41 42 43, 44 46 47
3. Level of SUI	33, 49, 52, 66 42 **(** 34, 36, 39, 50, 67, 68 **)** 47 46 45, 53 69, 56 38	3. Ethical Practice	33, 35, 41, 45 42 **(** 39, 43, 69 **)** 36, 49, 50, 51, 52, 53, 56, 67, 68	3. Enactment	33, 52 47 **(** 35 **)** 43, 45 47, 50, 51 **(** 49, 52, 53 **)** 40 41, 44 50 36, 55 42 45 **(** 39 **)**
4. Recruitment	33, 41, 49, 52, 66 **(** 35 **)** 42 **(** 39, 67 **) (** 36 **)** 34, 50, 68 47 46 53 45 69 56 38	4. Methods	33, 34, 35, 41, 45, 46 42 **(** 39, 43, 69 **) (** 36, 47, 49, 50, 51, 52 **)** 38 **(** 67 **)** 53, 56, 66 68	4. Appraisal	33, 43, 51, 52 47 33, 50, 52 56 38, 40
5. Training	39, 66 50 69	5. Partnership and Collaboration Data for this construct were extracted from: Theme 1 Socio‐political context or drivers Theme 2 Definition Theme 3 Level of SUI	33, 34 35, 41, 45, 46 42 **(** 39, 43, 69 **)** 36, 47, 50, 51, 52 38 **(** 67 **)** 56, 68 44 37, 40, 55		
6. Ethics	33, 49, 52 35, 41 42 67 **(** 39 **) (** 36, 46, 50, 68 **)** 45, 53 69 56	6. Roles and responsibilities	33 **(** 34, 35, 41, 45, 46 **)** 42 **(** 39, 43, 69 **) (** 49 **)** 38, 47, 50, 51, 52 56, 66 68 44 37, 55 40		
7. Methodological considerations	33, 49, 52 35, 41 42 **(** 67 **) (** 36, 39, 50 **) (** 34, 68 **)** 47 46, 53 45 69 56 38	7. **Standard practice	33, 35, 41, 45, 46 39, 42, 43, 69 **(** 49 **)** 36, 50, 51 52, 67 53, 66 56		
8. Dissemination		8. Other	**(** 34, 35, 41, 45, 46 **) (** 39, 43, 69 **) (** 49 **)** 36, 50, 51 47, 52 38 **(** 67 **)** 66 53 56, 68		
9. Impact of SUI	33, 49, 52 35, 41 42 **(** 67 **)** 36, 39, 50 **(** 34 **)** 68 47 46 53 45 69 56 38				
10. Evaluation of SUI	52, 66 33, 49 41 **(** 35 **)** 42 **(** 67 **) (** 39 **) (** 36, 50 **)**				
11. Other	47, 68 46 53 45 69 56 38

Box 2 **Our working definition of first‐, second‐ and third‐order constructs (drawing on work of^63^, ^64^, ^65^)**

**Table:** 

First‐order constructs	Information extracted during the critical appraisal process of reviewing the evidence in the literature for Service User Involvement in Primary Care Research and Health Service Development	Interpretations of what the literature tells us about Service User Involvement in Primary Care Research and Health Service Development	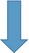
Second‐order constructs	Interpretation and collation of themes from first‐order constructs	Interpretations of what the literature tells us about Service User Involvement in Primary Care Research and Health Service Development	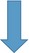
Third‐order constructs	The views and interpretation of the synthesis team expressed in terms of themes and key concepts and mapped onto four NPT constructs	Interpretations of what the literature tells us about Service User Involvement in Primary Care Research and Health Service Development	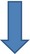

These first‐order constructs informed the development of our second‐order constructs through interpretation and collation of themes from first‐order constructs (see Table 3, Columns 3 and 4).

Whilst we were reviewing our second‐order constructs and the data contained therein, we were exploring the evidence from across all studies in the review to integrate this data into a synthesizing argument. Given our noted link between the practice of SUI and NPT's constructs, we developed a synthesizing argument around the theory's four constructs of coherence, cognitive participation collective action and reflexive monitoring (Table 3 shows how the data from each paper informed this final synthesis).

By mapping our first‐ and second‐order constructs onto these synthesizing NPT constructs in a sequential manner, we were representing the network of synthetic constructs and explaining the relationships between them with the aim of providing a more insightful formalized and generalizable way of understanding a phenomenon^29^ – in this case, the phenomenon of Service User Involvement in Primary Care Research and Health Service Development. Therefore, the synthesizing constructs of NPT were informed by data from across the second‐order constructs (which were developed from thematic analysis and interpretation of first‐order constructs).

In the main, the second‐order constructs which most informed the final stage of our synthesis and conclusions were partnership and collaboration, roles and responsibilities and power and control (See Table 4 below).

**Table 4:** Description of second‐order constructs – partnership and collaboration, roles and responsibilities, and control and power – informing the third‐order constructs

Theme name	Theme content
Partnership and Collaboration Subthemes Collaboration in health‐care delivery Collaboration in clinical consultation Collaboration in research	The data in this theme relate to partnerships and collaborations for research and health‐care projects. Data refer to the working relationship and style of working involved in partnerships and collaborations. Data also refer to working in a specialized way or with specialized roles within partnerships and collaborations The data in this theme relate to where collaboration has happened and there is evidence of what happens when collaboration and partnership are in place. Data that describe partnership with service providers, partnership between systems (socio‐political systems, health systems), communities and individuals were also included here. Data were included if they describe system‐level changes that are required for partnership and collaboration to occur
Roles and Responsibilities Subthemes Diverse research roles The ‘expert patient’ role Roles in clinical interventions	The data in this theme refer to particular roles or responsibilities that were defined and described in the research paper. The focus is on actions and interactions by stakeholders in the research These data relate more specifically to practice rather than rhetoric
Control and Power Subthemes The rhetoric of SUI Issues of equity and human rights Empowering research methods	Data coded in this theme refer to issues of service user control, or lack of, in health‐care settings or health‐care research. Emancipatory methods used in research studies or in clinical collaborations to readdress the balance of power are described. The process and implications of rebalancing power and regaining power are also discussed. Data also include references to equality in health‐care relationships and the levers and barriers to equity. The role that research can play in this power dynamic is more explicitly discussed. Examples include data where research brings about changed mindsets, surrenders power, or realigns control and power in relationships

The other themes generally described standard information about the conduct of the research typically contained in an academic write‐up of a peer‐reviewed paper, but they did not reveal anything specific about the topic of service user involvement itself. For example, all accounts of Ethical Practice referred to standard procedures of applying and receiving ethical approval. There were no data about specific ethical considerations that had to be taken into account to support/enhance the service user involvement dimension, for example, the development of training or mentoring to enhance service users’ capacity for co‐working and co‐governance.

Our findings are reported under the headings of definition, enrolment, enactment and appraisal below.

## Findings

The majority of papers were from the UK (*n* = 11). Four were from Ireland, three from the US, two from Australia and one each from Brazil, Canada, Finland, Germany, Sweden and The Netherlands.

### Definition

Only six papers included a definition of service user involvement.^33^, ^34^, ^35^, ^36^, ^37^, ^38^ All six definitions focused on the notion of SUI as involving partnership, collaboration, and notions of ownership and empowerment for service users. Thus, we inferred that in each case, the researchers were indicating strong aspirations for meaningful service user involvement. In the other papers, while authors did not provide an explicit definition, many stated that their project had been designed in response to policy imperatives to re‐balance power and control between those planning or delivering health services, and those who use the health services.

There were no data in any paper about whether definitions within or across stakeholder groups differed, so we could not determine whether the definitions were shared or not by those involved in the work.

### Enrolment

Several papers reported that the rationale for creating partnerships and collaborations was specifically to reform aspects of health‐care delivery by drawing on the experiences and perspectives of service users in research projects.^39^, ^40^, ^41^, ^42^, ^43^, ^44^ O'Reilly *et al*.'s aim^40^ was to gather drug users’ perspectives on how they are treated by services, and to assess drug users’ views of health services to change services. There were examples of collaborations that were initiated to improve clinical consultations, specifically, studies about shared decision making (SDM) which aimed to improve adherence, satisfaction with treatment and clinical outcomes.^45^ Other studies enrolled patients in SDM for depression treatment,^36^ and for the treatment of depression in patients with cancer^42^ and for cardiovascular risk management.^39^

Other partnerships and collaborations were initiated for the purposes of iterative testing and refinement of clinical tools with providers and patients.^46^ For example, Goodrich *et al*.^46^ sought patient input into the development of an Internet‐mediated walking programme to develop and evaluate an online interface and to monitor participant progress in the programme.

Partnership in health‐care service development was addressed by Peconi *et al*.^47^ in scoping activities that brought together key players and stakeholders in emergency and unscheduled care, strengthening commitment to the proposed research and development network. This was reported to be the first time in Wales that these groups were brought together across the emergency care system, to focus on research issues in this way.

Across studies overall, the key reported rationale for enrolment in research and health service developments was to bring the service user perspective to the work in hand. This is consistent with a vision of primary health care as one that stretches the boundaries of relationships beyond formal agencies and professionals, to include community representatives as collaborators to influence health‐care delivery, health‐care decisions and research.^48^ However, what is missing are data on *who* initiated the collaborations, and data about *what* strategies, if any, were employed to facilitate meaningful collaboration, as well as critical reflections on the nature of the partnerships or collaborations.

### Enactment

Service users reportedly held a diverse set of roles and responsibilities across the studies reviewed. The most common one was being asked to comment on study materials/proposed interventions, and often there is evidence of some changes to the projects as a result of their input. However, as reported above, despite the fact that most papers’ explicit or implicit definitions indicated strong aspirations for meaningful service user involvement, standard methodologies such as interviews and focus groups were generally employed.^35^, ^43^, ^45^, ^47^, ^49^, ^50^, ^51^, ^52^, ^53^, ^54^

There were some examples of innovative methods that enabled more meaningful involvement, and these were studies that were designed at the outset as participatory health research projects.^33^, ^40^, ^41^, ^44^, ^50^, ^52^, ^55^ Here, there was evidence of stronger congruence between the aims of sharing power and control and practicing emancipatory principles, and the methods employed. For example, de Brún and Du Vivier conducted a participatory learning and action (PLA) research study with homeless men to design an intervention using PLA timelines (designed to elicit accounts of life journeys and stories of personal breakthroughs). A visual representation of the meta‐analysis of their experiences was presented in a PLA matrix chart.^44^ Interestingly, participants *and* researchers generated data together on the topic of interest, shared each others’ experiences and perspectives, and completed a co‐analysis together. In the Alexander study, emancipatory actions during the research process supported ethnically diverse women to regain control of their health care and maintain equality over the course of five successive focus group meetings and in their subsequent interactions with primary care clinicians.^33^

Furthermore, in several of these participatory studies, it was evident that service users had sustained involvement in the project, with changing roles as per the project progression. Lindenmeyer *et al*.^50^ reported that service users shaped the direction of their work at the outset, and also assisted with recruitment, the development of questionnaires, analysis and dissemination activities. However, even where such efforts were made, an explanation of how a participatory research process can lead to empowerment or other similar positive health service outcomes was lacking, which begs the following questions: What actually happens in the research process that leads to these outcomes, and in what ways can researchers elucidate or report these processes?

Finally, it was interesting to note that in the majority of papers, reviewed participants are referred to as ‘patients’, which perhaps reflects notions of the ‘expert patient’, that is that patients have expertise by virtue of their experience of a particular condition or illness or health service utilization. In other cases, rather than using the term ‘patient’, the authors used terminology that emphasized the individual's or group's socio‐demographic identity, for example men,^51^ women^33^ or older people.^43^ Whether this difference in terminology is indicative of researchers’ attitudes towards the service user, which in turn may be reflective of power differentials within research or service development initiatives, is hard to say, but it may be worthy of further examination.

### Appraisal

Authors’ appraisals of their work were mostly positive. The most consistent claim made was that service users offered a unique and practical expertise that added credibility to the work with positive impacts on service delivery of research. Many authors reported that SUI added real‐world connection to their research,^43^, ^47^, ^51^, ^52^ and changed the mindsets of researchers.^50^

There were reported benefits for service users. For example, confidence and self‐knowledge increased,^33^ confidence in making health‐care decisions increased,^33^, ^56^ a sense of power increased,^33^, ^56^ and participants learned how to speak up and talk back.^33^, ^56^ Equality in the research process led to positive interactions^33^ and equality of interaction.^50^ Interestingly, only one paper^40^ provided data from service users directly to support these claims.

In contrast, negative outcomes were rarely reported, for example whether there had been frustrations, power struggles or disengagement by either the service users or health‐care workers/researchers. Few studies explored the problems or challenges of participation, including passive consumer roles and tokenism. A notable exception was Radermacher *et al*.^38^ who provided a critical analysis of the powerlessness of people with disabilities in the face of organizational structures and culture.

## Discussion

In this review, we focused on documented problems in the field of involvement in primary health‐care research and health‐care service development projects – problems of definition, enrolment, enactment and appraisal. We have critically interrogated conditions for implementation of meaningful involvement in primary care in primary health‐care research and health services development projects.

Our findings confirm rather than resolve the problem of definition. Only six papers in our sample provided an explicit definition to convey the meaning of the work they were doing. The definitions provided were typically in the introduction section, with references to existing literature. There were no empirical data about how different stakeholder groups involved in the project defined involvement. This limited the scope for our intended analysis of issues of definition vis‐à‐vis enrolment, enactment and appraisal within individual projects, and diminishes the scope for strong coherence in the field.

In terms of enrolment, based on these available data, we see an emphasis on policy imperatives to involve service users in primary care to share power and control. There is a sense that those involved in research and health‐care delivery projects believe it is right that they engage with stakeholders to follow policy imperatives, but less evidence that they believe it is worthwhile and valuable as *a way of working*. It was interesting to contrast the rhetoric about sharing power and control with the apparent gravitation to high ideals about meaningful involvement with the enactment of SUI. Much of what is reported reflects standard practice in health service research and health service development projects rather than evidence of a body of specialized practice that is committed to realizing such high ideals.

Notwithstanding the fact that there are ethical practices and scientific principles that are important across all kinds of research designs, we would argue that it is reasonable to expect to see *additional and/or creative* activities in this particular field. The purpose of such activities would be to enable service users to undertake roles and responsibilities that go beyond ‘having an input’. Service users would be enabled to create and participate in meaningful and on‐going partnerships and collaborations, which in turn would enhance the scope for sharing power and control. In this review, there were papers that claimed to have these aims but did not employ suitable methodologies to achieve those goals. For example, most papers reported involvement at only one point in time, echoing findings reported elsewhere by Brett *et al*.^9^ who found that user‐led or collaborative studies with users were more likely to demonstrate sustained involvement.

There are valuable examples in our data set of the specific considerations that some researchers reported in their efforts to enact more meaningful participatory approaches. Alexander^33^ describes her investigator role as providing a forum where analysis, reformulation and recognition of emancipatory interests could be supported and encouraged, and she outlines the use of a participatory group methodology to create such a context for women in the research process to experience empowerment. De Brún and Du Vivier describe their decision to generate data and share *their* life stories and turning points with the homeless men with whom they were engaged.^44^

Our finding about enactment echoes previous work, but we emphasize that a major finding from this review is that the balance of work in the field appears to be consultative rather than participatory.^21^ Moreover, there are significant gaps in the field that make it challenging to progress the realization of emancipatory ideals in this field.^7^ For example, the gap in knowledge about whether stakeholders have shared or differential understandings of the work in hand is problematic. Such ambiguities can cause frustrations and misunderstandings which become barriers to meaningful involvement.^12^ Worse, we know that repeated disappointments with research involvement among specific communities can accumulate, leading to research fatigue and resistance to partnerships and collaborations with university or health service personnel.^57^, ^58^, ^59^ This in turn affects the appraisal and reported outcomes of SUI for research and health service development projects.

Therefore, to advance our understanding and practice, it is important that issues of definition and expectations are made explicit, so that appraisals of outcomes can be fair and meaningful. A recent review of the benefits of participatory research^17^ carefully identified and explicated key characteristics of participatory practice, which enabled compelling conclusions to be drawn about the positive impact of such research approaches. This kind of attention to framing the specifics of practice in the field is important for expanding its evidence base. In time, it would be good to see an evidence base about different levels of involvement and their outcomes. Like Brett *et al*.^9^ we argue that poor reporting of impact acts ‘as a fog obscuring the real impact of PPI’ (p. 15).

Another key finding from this review is the observed emphasis in the papers on positive appraisal and impact. The exception in our review was Radermacher *et al*.'s critical analysis of the powerlessness of people with disabilities in the face of organizational structures and culture. It is interesting to note that the analysis of barriers to participation was not incidental or secondary to their research, but was in fact their stated objective.^38^ This explicit attention to problems is unusual in our sample of literature, where the emphasis is on ideals and notions of only *good* practice because of/during service user involvement initiatives. Arguably, this more critical stance is as real and warrants further scrutiny, particularly given recent findings from the PIRICOM Review^9^ which reported negative impacts in terms of personal impact, skill levels and knowledge levels and users feeling overburdened, not listened to and marginalized.

### Methodological critique

This review was based on a search of one platform database only. Arguably, we could have included other platforms. However, EBSCO offers a suite of more than 300 full‐text and secondary research databases, and our intention was not to conduct a comprehensive review of all published literature but to generate a purposeful sample that represented the range of practice in this field. Our iterative development of sampling parameters during the search phase was crucial for this.

Our critique of the literature is limited to single accounts of the studies in our review, which in turn are limited by the strict criteria for style and word count of academic journals. This may, of course, influence the nature of what is reported and may explain the emphasis on positive findings. We did not engage in chain referencing to identify additional papers about the studies in our sample, and this may have augmented or modified our analysis. However, we did note that during our search, there were no obvious examples of related papers; had there been, we would have included them. We only included grey literature from an Irish context and not from an international setting, and we do accept that there are additional and extended accounts of service user involvement in practice in reports and other documentation that may have provided detail missing from academic journal articles. However, we had a specific interest in reviewing literature that had been through peer review and had therefore been accepted by our peers as a form of service user involvement and representative of work in the field. Our review is influenced by our background as participatory researchers and our national contexts. We have endeavoured to be open and reflexive about that throughout the work and in this article.

Finally, this review is limited to papers published up to 2011. We acknowledge that there are relevant recent additions to the literature that have further enhanced our knowledge about co‐productions of knowledge with expert laity,^60^ experiential expertise^61^ and positive contributions to research, for example acquisition of new skills, knowledge and experience.^60^ The GRIPP checklist for reporting the practice of PPI^62^ is another valuable addition, and indeed, there are similar findings across our two studies. However, the additional contribution this review makes is its focus on analysing current *practice* to understand *implementation* of SUI in health research and health services development, and to make recommendations that will improve practice and the chances of *normalization*. Our use of NPT was appropriate for these aims. It helped us to ‘think through’ complex data and interrelated macro‐, meso‐ and micro‐level issues because we could organize them conceptually under NPT's four constructs, which enhances understanding.^28^

### Directions for future research and practice

It would be valuable to seek answers to the questions of definition, enrolment, enactment and appraisal by prospectively conducting multiperspectival fieldwork with stakeholders about their work together in specific projects. This would be a very effective way to explore shared and differential perspectives. We also recommend that primary care researchers publish an explicit account of their working definition of ‘service user involvement’, the process by which that was determined (i.e. whether it was in consultation with other stakeholders or not), and an explanation of their choice of methods in relation to that definition. Effectively, this practice should become part of the repertoire of practice and reporting procedures by researchers engaged in the field of service user involvement to augment the evidence base, encourage more methodological innovation and enable robust appraisals of work that is undertaken.^48^, ^62^

## Conclusion

Following Normalization Process Theory, the likelihood that service user involvement becomes a routine and normalized way of working in health‐care settings relies on the four problems of definition, enrolment, enactment and appraisal being resolved. It is necessary to encourage explicit reporting of definitions employed, methodological innovation to enhance co‐governance and dissemination of research processes as well as findings. This will augment the evidence base about current practice and improve normalization of meaningful involvement.

## Sources of funding

This project was funded by the Health Research Board, Dublin, Ireland.

## Conflict of interest

The authors declare no conflict of interests.
